# Development of a novel information and communication technology system to compensate for a sudden shortage of emergency department physicians

**DOI:** 10.1186/s13049-017-0347-3

**Published:** 2017-01-23

**Authors:** Kumiko Tanaka, Taka-aki Nakada, Hiroshi Fukuma, Shota Nakao, Naohisa Masunaga, Keisuke Tomita, Yosuke Matsumura, Yasuaki Mizushima, Tetsuya Matsuoka

**Affiliations:** 1 Senshu Trauma and Critical Care Center, 2-23 Rinku Orai Kita, Osaka, 598-8577 Japan; 2 Department of Emergency and Critical Care Medicine, Chiba University Graduate School of Medicine, 1-8-1 Inohana, Chuo, Chiba, 260-8677 Japan

**Keywords:** Mobile phone, Cloud server, Mail, Information sharing, Trauma, Night, Mass casualty, Life threatening, Critical care

## Abstract

**Background:**

A sudden shortage of physician resources due to overwhelming patient needs can affect the quality of care in the emergency department (ED). Developing effective response strategies remains a challenging research area. We created a novel system using information and communication technology (ICT) to respond to a sudden shortage, and tested the system to determine whether it would compensate for a shortage.

**Methods:**

Patients (*n* = 4890) transferred to a level I trauma center in Japan during 2012–2015 were studied. We assessed whether the system secured the necessary physicians without using other means such as phone or pager, and calculated fulfillment rate by the system as a primary outcome variable. We tested for the difference in probability of multiple casualties among total casualties transferred to the ED as an indicator of ability to respond to excessive patient needs, in a secondary analysis before and after system introduction.

**Results:**

The system was activated 24 times (stand-by request [*n* = 12], attendance request [*n* = 12]) in 24 months, and secured the necessary physicians without using other means; fulfillment rate was 100%. There was no significant difference in the probability of multiple casualties during daytime weekdays hours before and after system introduction, while the probability of multiple casualties during night or weekend hours after system introduction significantly increased compared to before system introduction (4.8% vs. 12.9%, *P* < 0.0001). On the whole, the probability of multiple casualties increased more than 2 times after system introduction 6.2% vs. 13.6%, *P* < 0.0001).

**Discussion:**

After introducing the system, probability of multiple casualties increased. Thus the system may contribute to improvement in the ability to respond to sudden excessive patient needs in multiple causalities.

**Conclusions:**

A novel system using ICT successfully secured immediate responses from needed physicians outside the hospital without increasing user workload, and increased the ability to respond to excessive patient needs. The system appears to be able to compensate for a shortage of physician in the ED due to excessive patient transfers, particularly during off-hours.

## Background

Emergency department (ED) crowding is a significant concern, with increased risk of harm to patients [1–3]. Sufficient healthcare staff resources to meet patient needs are essential to preserve quality of care [4]. Full resource availability is optimal, but increases medical costs. Thus, matching resources to patient needs is a key issue to be addressed. However, patient needs in the ED are not always constant and predictable [5–7].

Medical resources tend to decrease at night or on weekends [8, 9]. The impact of off-hour effects can alter clinical outcomes in ED patients [4, 10–16]. To compensate for a shortage of physician resources, an off-hour, on-call physician system has often been used [17]. However, the number of on-call physicians is also limited due to cost and/or physician workload; the on-call physician system cannot always compensate for mismatching. If mismatching occurs in the on-call physician system, a longer time and more labor may be required to determine physician availability on an individual basis.

Recent advances in information and communication technology (ICT) have brought significant changes in both daily life and medicine [18–20]. For instance, ICT shortened response times of layperson for cardiopulmonary resuscitation in out-of-hospital cardiac arrest [21]. Advantages of ICT include: (1) simultaneous rapid transmission of information to multiple individuals via Internet line/cloud server, (2) processing of transmitted data by software in the cloud server instead of by the end-user (for instance, web-based software can automatically transform data into easily recognizable formats and updates at all times), and (3) real-time information sharing among multiple individuals. These advantages can be utilized to develop a system to compensate for a shortage of physician resources due to suddenly overwhelming patient needs without increasing user workload. However, to our best knowledge, such an ICT system has not yet been developed.

In the present study, we developed a novel system to respond to a sudden shortage of physician resources due to overwhelming patient needs in the ED. We tested a hypothesis that this system secures physician resource and improves the response to the multiple casualties in a level I trauma center in Japan. We primarily evaluated whether the activation of the system secured the necessary number of physicians without using other means, such as a phone or pager.

## Methods

### Study design, patients, and hospital

The current observational study was prospectively conducted. We created a completely new system using ICT to request immediate responses from physicians outside the hospital when an attending physician detected a potential risk of excessive patient needs that could affect the quality of care and clinical outcomes. The system was introduced to the Senshu Trauma and Critical Care Center in Japan in September 2013. The current study comprised 4890 patients with a high percentage of life-threatening conditions who were transferred to the ED of the tertiary center between September 2012 (1 year before system introduction) and August 2015 (2 years after system introduction), and included 3559 patients during the 2-year period after system introduction.

The Senshu Trauma and Critical Care Center is a level I regional trauma center that provides advanced trauma and critical care 24/7 and exclusively receives patients with life-threatening emergencies. Emergency patients without a life-threatening condition are transferred to a different ED in a connecting secondary care center. In one year of the study period (2014), a total of 1873 patients were received by ambulance and 568 emergency surgeries and 105 endovascular examinations/treatments were performed. Twenty-four trained trauma and critical care physicians worked in the center in day and night shifts (day, 8:00–17:00; night, 17:00–8:00). During nights or weekends (Saturday and Sunday), 4 physicians work in the hospital, and 7 predetermined on-call physicians are on standby outside the hospital for emergencies (on-call physicians: acute care surgeons for general surgery [*n* = 3], neurosurgeons [*n* = 2], and interventional radiologists [*n* = 2]). These predetermined on-call physicians were contacted from the center by phone when needed, and proceeded to the center both before and after system introduction. In addition to this basic system of predetermined on-call physicians during nights or weekends, we introduced the new system.

The study center is located in an urban area in Osaka, Japan, covering about 1 million residents within a 40-km radius. Neighborhood general medical centers providing emergency and critical care are 50 km away. The attending physician can ask emergency medical services (EMS) personnel to transfer a patient to a neighborhood center in the event of excessive patient load and a shortage of physician. Thus, in the present study, we evaluated the probability of multiple casualties among total casualties transferred to the ED of the study center, as an indicator of the ability to respond to excessive patient needs.

### New ICT system

**Fig. 1:**
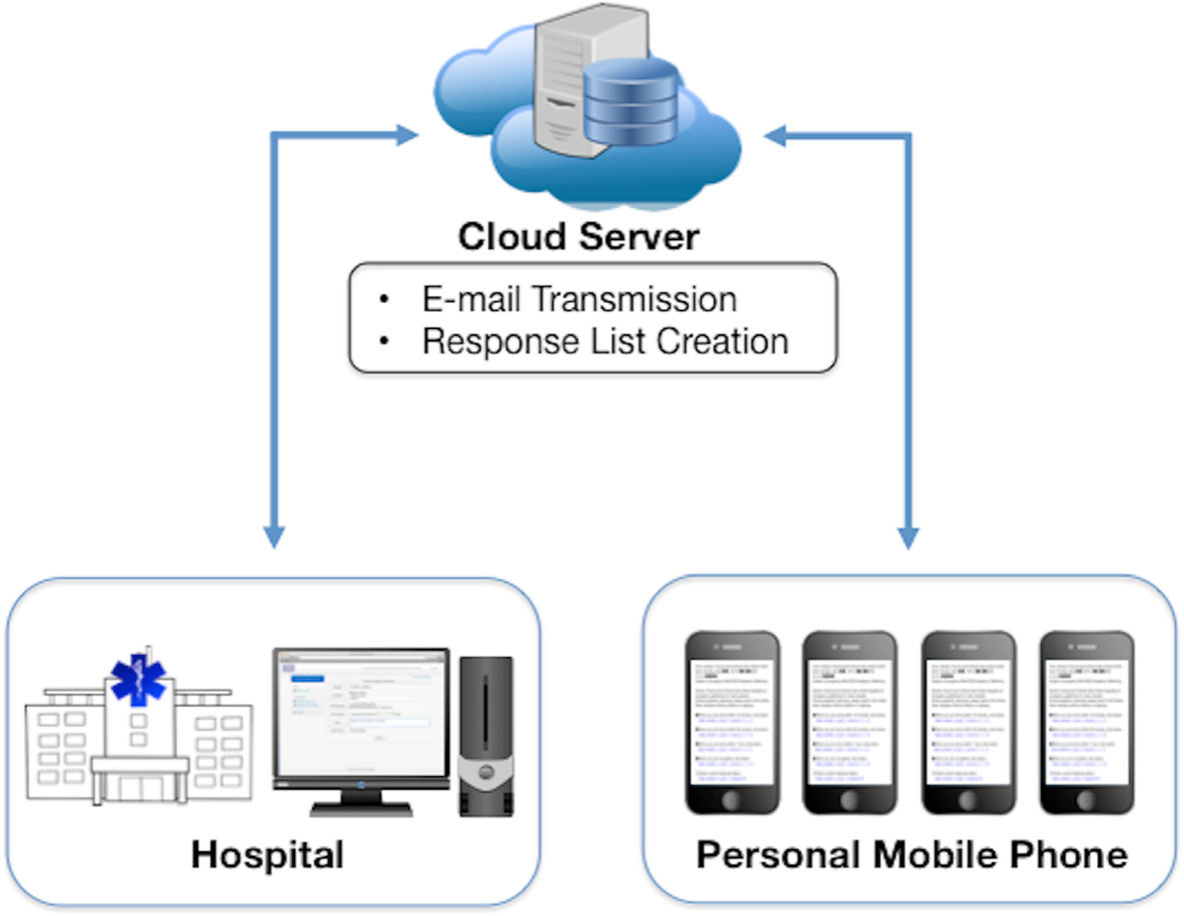
Schematic diagram of data flow in the newly developed system. A requester in the hospital activates the system by filling in a request form. The request is securely transferred to a cloud server via Internet line. The cloud-based software automatically creates an e-mail based on the content of a request, and sends the e-mail to physicians’ personal mobile phones. Responses from physicians outside the hospital are transferred to the cloud server, where a list of current response status is created and updated at all times, and shared in real-time among physicians both inside and outside the hospital

**Fig. 2:**
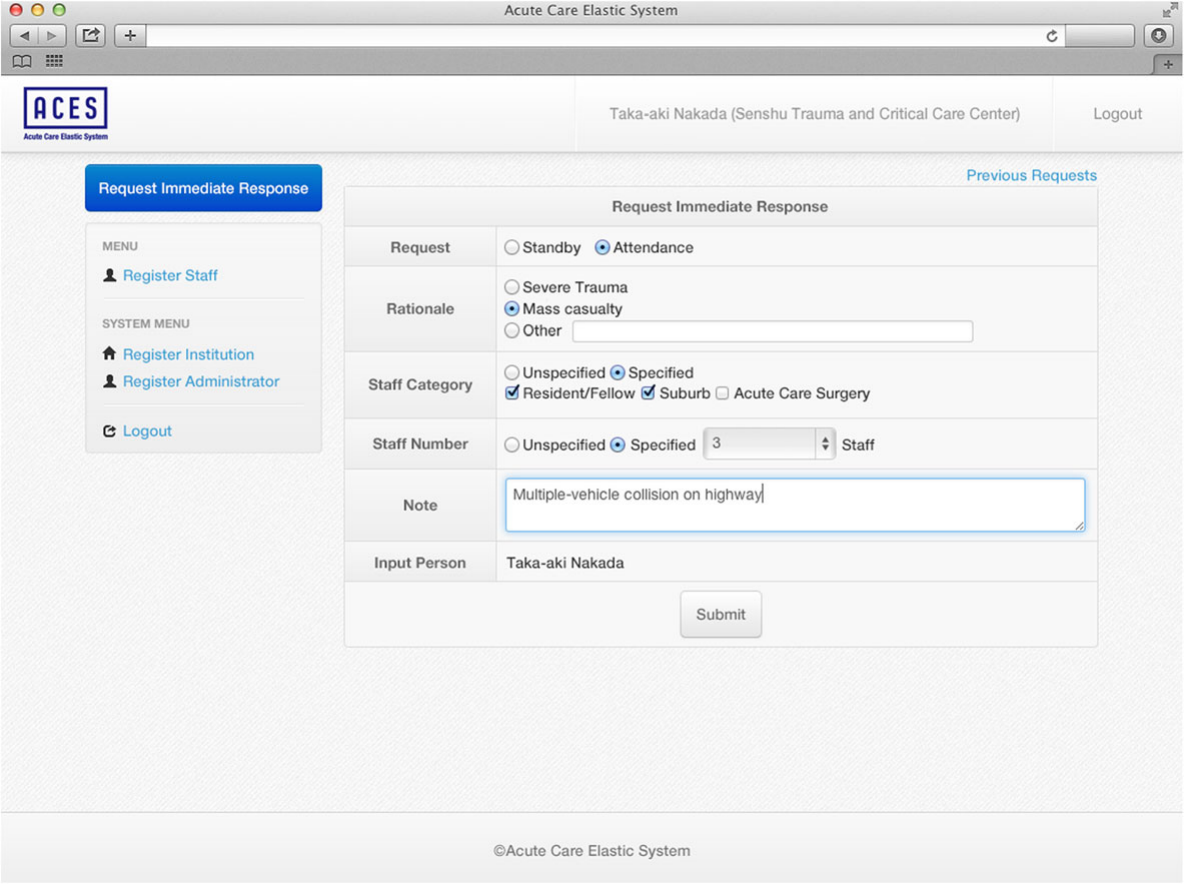
Input screen for request. The requester selects 4 input items by button input including categories of request, rationales for request, and needed type and number of physicians. To enable inclusion of key information in the request, two text input fields in the section (rationale and note) were provided

We created the novel system to compensate for a shortage of physicians due to excessive patient transfers using ICT (Fig. 1; co-developed with Cereja Technology Co. LTD, Tokyo, Japan). The system is straightforward. A requester activates the system by filling in a form (Fig. 2). The request form includes 4 input items: (1) category of request (standby or attendance), (2) rationale for request (severe trauma, mass casualty, other), (3) required staff category (resident/fellow, living in the suburbs of hospital, acute care surgeon [multiple selections] or unspecified), and (4) number of physicians needed. To enable rapid completion, we adopted button inputs for these items. In addition, we provided text input fields in the section for rationale and notes to allow inclusion of key information in the request (Fig. 2).

**Fig. 3:**
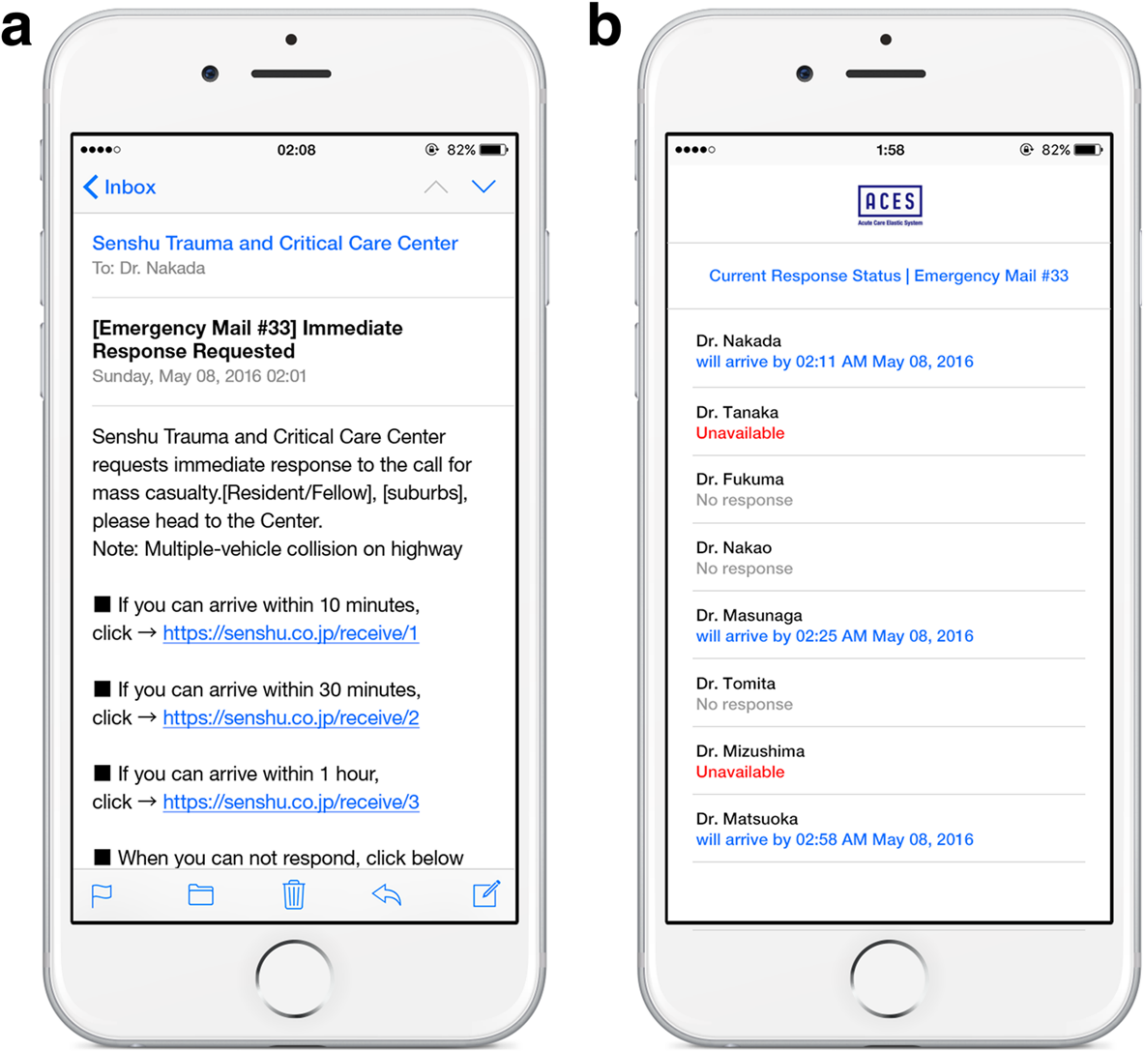
**a**. Received mail text in mobile phone of physician outside hospital. **b**. List of current response status in mobile phone

After submission of the request, the software securely transfers the request from the hospital to a cloud server via an Internet line (Fig. 1). The cloud-based software automatically creates an e-mail based on the content of the request, and sends the e-mail to physicians’ personal mobile phones within a few seconds. Physicians who receive the e-mail can answer the request with a single click in the e-mail according to their status (Fig. 3a). Based on the answers that are clicked and transferred to the cloud server (Fig. 1), the cloud-based software automatically generates and updates a physician list with current response status at all times (Fig. 3b), and shares information in real time between physicians both inside and outside the hospital. It is possible to be off-duty in the system.

The request can be sent from any device such as smartphone or tablet devices (iOS, Android), laptop or desktop computers (Windows, Mac) when connecting internet via cloud platform. Physicians receives the request as an email on their devices such as cellular phones, smartphones or computers without installation of specific application or OS update.

### Statistical analysis

We first assessed patient data using the novel system after its introduction over a 2-year period. Data were presented as mean and standard deviation. As a primary analysis to evaluate the utility of the system, we evaluated whether its activation secured the needed physicians without using other means such as phones (phones in the basic physician on-call system were excluded) or pager, and calculated fulfillment rates using the system. As a secondary analysis, we tested for a difference in the probability of multiple casualties among total casualties transferred to the ED of the study center before and after system introduction, using a chi-square test. Analyses were performed using SPSS statistical software (SPSS, version 20, Armonk, NY, USA).

## Results

During the 2 years after introduction of the system, 3559 patients with a high probability of life-threatening conditions were transferred to the ED of the trauma and critical care center (Table 1). Of the 3559 patients, 997 (28.0%) received initial care concurrent with another critical emergency patient(s) (2 patients at a time [*n* = 868] and 3 patients or more at a time [*n* = 129]).

**Table 1:** Data on patients and the novel emergency mail system

Patient data	
Admitted patients -n	3559
Timing of admission -n (%)	
Daytime -n, total (per hour)	1573 (174.8)
Night -n, total (per hour)	1986 (132.4)
Weekday -n, total (per day)	2515 (503)
Weekend -n, total (per day)	1044 (522)
Multiple simultaneous patients -n (%)	997 (28.0)
2 patients	868 (24.4)
≥ 3 patients	129 (3.6)
System data	
Category of request	
Standby	12
Attendance	12
Timing of activation -n	
Daytime on a Weekday	1
Off-hours	23
Weekend	12
Night	19
During emergency surgery	14
Rationale for activation -n	
Mass casualty	15
Severe trauma	4
Others	5
Attendance request	
Pre-existing physicians -n	5.1 ± 1.6
Pre-existing patients -n	2.5 ± 1.4
Requested physicians -n	2.2 ± 0.8
Attended physicians -n	2.0 ± 1.2
Cancelled physicians -n	2.0 ± 2.0
Arrival time -min	21 ± 9.0
Fulfillment rate -%	100 (12/12)

Data from September 2013 to August 2015 are shown

Daytime, 8:00–17:00; Weekend, Saturday and Sunday

Fulfillment rate, (Number of cases for which the new system achieved sufficient physician attendance) / (Total number of emergency attendance requests) × 100

Data are mean and standard deviation

The system was activated once a month on average (24 times in 24 months). The 24 activations included 12 standby requests and 12 attendance requests. Of the 24 activations, 23 (95.8%) occurred during weekends or nights. The primary rationale for activation was mass casualty (15 of 24 activations) (Table 1). For instance, an attending physician received a request at 02:00 from EMS personnel to transport 3 severely injured patients (2 unresponsive patients with severe head injuries and 1 patient with a severe abdominal injury) and 2 patients injured in a multiple vehicle collision; the system was activated. The system successfully obtained immediate attendance of physicians from outside the hospital to provide advanced trauma care including emergency surgery. In addition, the system was activated 14 times during emergency surgery. For instance, in the middle of an emergency operation for a severely injured multiple trauma patient involving 7 physicians (4 night shift physicians and 3 on-call physicians [2 interventional radiologists and a neurosurgeon]) at 22:00, EMS personnel requested permission to transfer 2 severely injured patients with open lower extremity fractures due to a tandem motorcycle accident; the system was activated and successfully obtained immediate responses from the needed number of physicians outside the hospital.

In 12 activations for attendance requests, an average of 5.1 pre-existing physicians provided emergency advanced care for an average of 2.5 pre-existing ED patients (Table 1). An average (± standard deviation) of 2.2 ± 0.8 physicians were requested to attend via the system; 2.0 ± 1.2 physicians immediately responded with arrival time of 21 ± 9.0 min. An average of 2.0 ± 2.0 physicians cancelled the request. In these activations, the system successfully secured the needed physicians without using other means, and the fulfillment rate, which was a primary outcome variable of the present study, was 100% (Table 1).

**Fig. 4:**
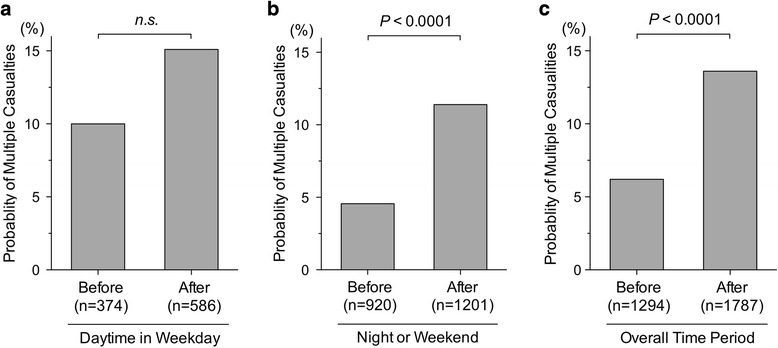
Probability of multiple casualties with a high likelihood of a life-threatening condition before and after system introduction. The probability of multiple casualties with a high likelihood of life-threatening conditions before and after system introduction was not significantly different during daytime weekday hours (before vs. after, 10.0% vs. 15.1%, *P* = 0.062) (**a**). However, during nights or weekends, the probability of multiple casualties with a high likelihood of life-threatening conditions after system introduction was significantly higher compared to before system introduction (before vs. after [nights or weekends], 4.8% vs. 12.9%, *P* < 0.0001) (**b**), as well as in the overall time period (before vs. after, 6.2% vs. 13.6%, *P* < 0.0001) (**c**). *P* value was calculated using a chi-square test

We next tested for a difference in probability of multiple casualties among total casualties transferred to the center, as an indicator of ability to respond to excessive patient needs, before and after system introduction. There was no significant difference in the probability of multiple casualties during daytime weekday hours before and after system introduction (before vs. after [daytime weekday hours], 10.0% vs. 15.1%, *P* = 0.062) (Fig. 4a); however, on nights or weekends, the probability of multiple casualties after system introduction was significantly higher than that before system introduction (before vs. after [nights or weekends], 4.8% vs. 12.9%, *P* < 0.0001) (Fig. 4b). On the whole, the probability of multiple casualties increased more than 2 times after system introduction (before vs. after [overall time period], 6.2% vs. 13.6%, *P* < 0.0001) (Fig. 4c).

## Discussion

In the present study, we developed a novel ICT system to compensate for a sudden shortage of physicians due to excessive patient needs in the ED. The system successfully secured the needed physicians for emergency advanced care without using other means in all activations for attendance. Moreover, after system introduction, the probability of multiple casualties increased more than 2 times.

Medical resources including physicians in the ED tend to decrease during off-hours [8, 9, 16, 22, 23]. Consistent with this, the system was mostly activated during off-hours. Patients who presented during off-hours had increased mortality from acute myocardial infarction (AMI), stroke, out-of-hospital cardiac arrest, urgent surgeries, and trauma [10–15], which highlighted the importance of developing a system to rapidly adjust for unpredictable imbalances, in order to preserve the quality of care [4]. An on-call physician system using a phone or pager has been widely used to address such imbalances, particularly for emergency patients during off-hours. However, the limited number of on-call physicians could remain a problem. Additionally, oral communication by phone, which is a typical contact means, has several disadvantages. Phones cannot easily contact multiple physicians simultaneously, thus reducing the likelihood of obtaining attendance from multiple physicians, or requesting standby at an early phase when information is preliminary. Thus, a novel method was needed to achieve more rapid and effective adjustment of imbalances, which was the motivation of the present study.

The new system has several properties. The first is speed and simplicity. System activation is rapid through simple and minor operations (Fig. 2), and the receiver can rapidly respond with a single click (Fig. 3). The second property is bidirectionality. The system sends a request from the hospital and receives responses from physicians outside the hospital. The third property is real-time sharing of information in easily recognizable formats. Responses regarding availability and predicted arrival time from physicians outside the hospital were automatically transformed to current response status in easily a recognizable format, updated at all times, and shared in real-time. Fourth, the system has a standby function, which enables the requester to report a potential risk at an early phase, with uncertain or preliminary information. This can help achieve rapid attendance in response to a subsequent request. Fifth, it has the function of automatic canceling. To avoid unnecessary attendance, when the needed number of physicians respond and head toward the hospital, the system automatically cancels the activation by sending an e-mail. Fifth, it repeatedly sends e-mail requests after activation (e.g., every 5 min), to prevent notification failure. These properties may have contributed to the successful fulfillment rate in the study.

Delayed initiation of treatment worsens clinical outcomes in AMI, stroke, surgery, and trauma [24–29]. We created the present system in order to achieve immediate responses, as a study using a ready-made messenger application for mobile phone (WhatsApp) showed decreased door-to-balloon time in patients with suspected AMI who presented to a rural ED without intervention capability and required transfer to a tertiary cardiac center [18].

In the preset study, we found increased probability of multiple casualties with a high likelihood of life-threatening conditions after introducing the system, particularly during off-hours. Thus, the system may improve the ability to respond to sudden excessive patient needs in multiple causalities with pre-existing physician resources.

This study has several limitations. First, it is a single-center study. Further studies involving multiple centers would strengthen the study results of a successful fulfillment rate and increased ability to respond to excessive patient needs. Second, the users of the system did not include all health care staff but only the physicians in the study. Third, during the study period, we did not experience massive patient needs, as in a large-scale disaster. Further development involving all healthcare staff may enable coverage for a major incident with a large number of patients.

## Conclusions

We developed a novel ICT system to respond to an unexpected shortage of physician resources due to overwhelming patient needs in the ED. The system successfully secured immediate responses from needed physicians outside the hospital, and increased the ability to respond to excessive patient needs. The ICT system may be useful in compensating for a shortage of physician in the ED due to excessive patient transfers, particularly during off-hours.
